# Call for caution on alleged tick-borne pathogens in blood-fed ticks from animals across nine Asian countries

**DOI:** 10.1128/spectrum.00507-25

**Published:** 2025-05-23

**Authors:** Carlos Ramiro Silva-Ramos, J. Manuel Matiz-González, Álvaro A. Faccini-Martínez

**Affiliations:** 1 Grupo de Enfermedades Infecciosas, Departamento de Microbiología, Facultad de Ciencias, Pontificia Universidad Javeriana, Bogotá, Colombia; 2 Molecular Genetics and Antimicrobial Resistance Unit, Universidad El Bosque 28009, Bogotá, Colombia; 3 Servicio de Infectología, Hospital Militar Central 150243, Bogotá, Colombia; 4 Facultad de Medicina, Universidad Militar Nueva Granada 28019, Bogotá, Colombia; Central Texas Veterans Health Care System, Temple, Texas, USA

## LETTER

We have read with great interest the manuscript by Byun et al. (1), “Detection of tick-borne pathogens in blood-fed ticks from animals across nine Asian countries,” published in Microbiology Spectrum journal, in which a metagenomics analysis was performed using next-generation sequencing technologies to analyze infectious agents, including viruses, bacteria, and protozoa, harbored by blood-fed ticks collected from different animal species across nine Asian countries (1).

We believe that taxonomic classification should be done with great caution, and the methodology used for this purpose must be applied carefully, as not all methods can confirm the presence of a microorganism and identify it at the species level. In the study performed by Byun et al. (1), the methodology employed was a shotgun metagenomics approach, analyzing reads of 200 bp or longer for assigning viral species. Although these reads were directly annotated against the NCBI database (>90% identity), this high identity does not provide sufficient evidence regarding the coverage of the identified viral genome. This lack of full genome context makes species-level classification uncertain (2, 3). For bacterial community profiling, a metabarcoding approach using the V3-V4 region of the 16S rRNA gene was performed (4, 5). This approach targets the highly conserved 16S rRNA gene, which contains hypervariable regions (V1-V9) that distinguish diverse taxonomic groups. Some next-generation sequencing techniques (e.g., Illumina MiSeq platform) analyze specific variable regions (e.g., V3-V4 or V4) as taxonomic markers, providing a cost-effective option but limiting species-level resolution (6). Even with full 16S rRNA sequencing, achieving accurate species identification remains a challenge (7, 8).

While these methods are highly useful and very effective for characterizing the microbial community in different samples, relying on them to identify specific species may result in incorrect reports and interpretations, which is probably occurring in the study performed by Byun et al. A great number of pathogens have been identified through this approach, all of them are described in Table 2 (1). Although many of these reports raise doubts and questions regarding the presence in blood-fed ticks from Asia, one of them is our biggest concern, which is the report of the presence of Guanarito virus (GTOV), the etiological agent of Venezuelan hemorrhagic fever (VHF) in ticks collected from birds from Japan. To the best of our knowledge, GTOV is a geographically restricted pathogen to a 9,000 km^2^ area in the southwestern region of Venezuela, mainly in Portuguesa and Barinas states, where it is known to be endemic, causing active cases of VHF. Despite a few studies performed in nearby regions such as Colombia, GTOV and active cases of VHF have not been identified anywhere else outside the endemic region in Venezuela (9). This seems to occur apparently due to the geographic distribution of its only known recognized natural reservoir, *Zygodontomys brevicauda* (Alston’s cotton rat). This species is the only one identified with the ability to control GTOV infection without eliminating it, resulting in chronic viremia and persistent long-term shedding of infectious viral particles in oropharyngeal secretions and urine (10, 11). Moreover, the distribution of *Z. brevicauda* is limited to certain countries in Central and South America (12).

We believe that several pathogens may be more widespread than previously thought, such as scrub typhus, which was once limited to the “tsutsugamushi triangle” but has recently been found in areas like southern Chile (13). However, studies proving this must be accurate and backed by sufficient scientific evidence to avoid incorrect reports.

## EDITOR'S NOTE

**Ed. Note:** The authors of the original article declined to respond.
